# Failure-mode-oriented design of cathode materials for practical lithium-ion batteries: from crystal stability to electrode-level durability

**DOI:** 10.3389/fchem.2026.1873892

**Published:** 2026-07-15

**Authors:** Xianzheng Liu, Feng Li, Bangsheng Yin, Guangyuan Wang, Haotian Wu

**Affiliations:** 1 College of Mechanical Engineering, Shandong Huayu University of Technology, Dezhou, Shandong, China; 2 Department of Mechanical and Manufacturing Engineering, Faculty of Engineering and Built Environment, Universiti Kebangsaan Malaysia, Selangor, Malaysia

**Keywords:** cathode materials, high-voltage cathodes, interfacial stability, lithium-ion batteries, oxygen redox, particle cracking, practical electrodes

## Abstract

Cathode materials remain the primary determinant of the energy density, voltage output, lifetime, safety, and cost of lithium-ion batteries. Although conventional reviews usually classify cathodes according to crystal structures or chemical compositions, practical battery degradation is rarely governed by structure alone. Instead, capacity fading, impedance growth, oxygen loss, transition-metal dissolution, interfacial parasitic reactions, and particle cracking usually occur simultaneously and are strongly coupled with electrode processing and operating conditions. This review discusses lithium-ion battery cathode materials from a failure-mode-oriented perspective, covering high-voltage LiCoO_2_, spinel LiMn_2_O_4_, olivine LiFePO_4_, and Ni-rich layered oxides. Rather than simply summarizing individual modification methods, it emphasizes how doping, coating, surface reconstruction, morphology regulation, and gradient design address specific degradation pathways. Particular attention is paid to the transition from material-level optimization to practical-cell durability, including electrolyte-dependent cathode–electrolyte interphase formation, cathode–anode crosstalk in full cells, thick-electrode transport, high-voltage interface compatibility, and chemo-mechanical stability. Finally, future directions are proposed for developing cathodes that combine high capacity, long cycle life, scalable processing, and practical safety.

## 1 Introduction

Lithium-ion batteries have become one of the most successful electrochemical energy-storage technologies because they combine relatively high energy density, long cycle life, and mature manufacturing compatibility (Yoo et al., 2014). After several decades of development, the basic working principle of lithium-ion batteries is well established: lithium ions shuttle between the cathode and anode during charge and discharge, while electrons travel through the external circuit to deliver electrical energy (Goodenough and Park, 2013). However, the apparent simplicity of this rocking-chair mechanism hides a complicated set of degradation processes. In real cells, lithium-ion transport, electronic conduction, electrolyte decomposition, transition-metal migration, gas release, stress accumulation, and electrode porosity evolution are strongly coupled.

Among all cell components, the cathode is especially important because it largely determines the cell voltage, accessible capacity, energy density, safety margin, and cost. Traditional cathode studies often begin with crystal-structure categories, such as layered LiCoO_2_ and Ni-rich oxides, spinel LiMn_2_O_4_, and olivine LiFePO_4_. This classification is useful, but it can also make different cathode families appear more separated than they actually are. In practical cells, many cathodes fail through similar pathways: unstable lattice oxygen at high voltage, surface reconstruction, resistive cathode–electrolyte interphase growth, metal dissolution, sluggish ion transport, and mechanical fracture (Cabana et al., 2018; Dong and Li, 2022). These shared failure modes suggest that cathode research should move from material-by-material optimization toward degradation-pathway control.

The electrolyte and separator also play essential roles in cathode operation. Electrolytes affect the stability of the cathode surface and the formation of interfacial films, while separators influence ionic transport, thermal behavior, and safety (Lagadec et al., 2019; Tan et al., 2023). Therefore, the future development of cathode materials should not only focus on raising theoretical capacity, but also on maintaining structural and interfacial stability under realistic operating conditions. This is particularly important for high-voltage LiCoO_2_, high-Ni layered oxides, and thick LiFePO_4_ electrodes, where performance is increasingly limited by coupled interfacial and mechanical issues rather than by intrinsic capacity alone.

This review therefore discusses cathode materials from a failure-mode-oriented perspective. The key question is not simply “which cathode has the highest capacity,” but “which instability limits each cathode under practical conditions, and how can material design suppress it?” Importantly, suppressing intrinsic material degradation alone is insufficient for practical batteries, because cathode performance is ultimately determined by the coupling between particle-level stability and electrode-level factors, including loading, porosity, conductive network, and interfacial compatibility. Based on this logic, high-voltage LiCoO_2_ is considered as a model system for oxygen and interface stabilization; LiMn_2_O_4_ is discussed as a spinel framework limited by Mn dissolution and Jahn–Teller distortion; LiFePO_4_ is analyzed from the viewpoint of transport-network construction and thick-electrode utilization; and Ni-rich layered oxides are reviewed as high-energy cathodes requiring simultaneous control of cation disorder, oxygen activity, and particle cracking.

Recent studies also show that cathode failure modes cannot be assigned to cathode chemistry alone. Under high-voltage operation, electrolyte oxidation can generate unstable CEI species, gaseous products, and HF-containing byproducts, which further accelerate surface reconstruction, transition-metal dissolution, and impedance growth. Dissolved Ni, Mn, or Co. species may subsequently migrate to Li-metal or graphite anodes, where they catalyze parasitic reactions, promote resistive SEI growth, and contribute to lithium-inventory loss or anode slippage. Therefore, half-cell results using excess Li metal should be interpreted cautiously, because they may mask lithium loss and anode-side degradation. A failure-mode-oriented review should therefore connect cathode surface chemistry with electrolyte formulation, anode crosstalk, and full-cell validation under realistic areal loading, electrolyte amount, and operating voltage.

**FIGURE 1:**
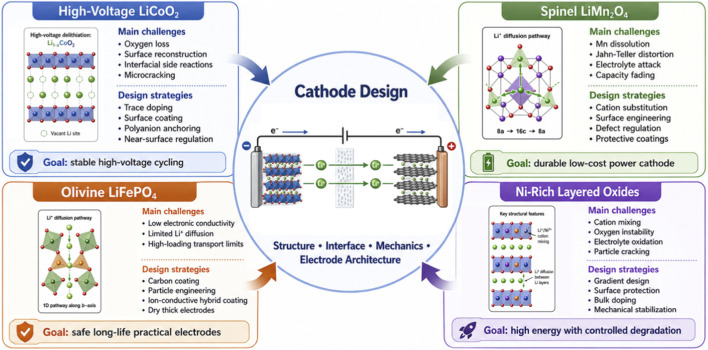
Failure-mode-oriented design framework for practical lithium-ion battery cathode materials. The framework also highlights recent design directions, including single-crystal and entropy-stabilized Ni-rich cathodes, ALD-derived or surface-reconstructed protective layers, electrolyte-compatible CEI construction, and thick-electrode architecture engineering. These strategies connect atomic-level stabilization, surface/interphase regulation, particle-level mechanical durability, and electrode-level transport optimization.

To clarify the logic of this review, Figure 1 presents a failure-mode-oriented design framework for representative lithium-ion battery cathode materials. High-voltage LiCoO_2_ is mainly limited by oxygen loss, surface reconstruction, and microcracking; spinel LiMn_2_O_4_ is strongly affected by Mn dissolution and Jahn-Teller distortion; olivine LiFePO_4_ requires improved electron/ion transport and thick-electrode utilization; and Ni-rich layered oxides suffer from cation mixing, oxygen instability, surface reconstruction, and particle cracking. Accordingly, cathode design should integrate atomic-level regulation, functional interphase construction, chemo-mechanical stabilization, and practical electrode architecture engineering rather than relying on a single modification strategy.

## 2 High-voltage LiCoO_2_: Stabilizing lattice oxygen and cathode surface chemistry

LiCoO_2_ remains a benchmark layered cathode because of its high tap density, high working voltage, and mature processing route. Its layered α-NaFeO_2_-type structure provides a clear lithium diffusion pathway, and its electrochemical behavior has been studied for decades. However, when the charging voltage is increased beyond the conventional range, deeper lithium extraction destabilizes the host lattice and intensifies surface reactions (Lin et al., 2024). The major challenge for high-voltage LiCoO_2_ is therefore not the lack of theoretical capacity, but the difficulty of extracting more lithium while preventing oxygen loss, phase transition, interfacial side reactions, and microstructural damage.

At high voltage, the hybridization between Co. 3 d and O 2p orbitals becomes increasingly important. Excessive oxygen participation can trigger oxygen escape, surface reconstruction, and irreversible structural changes. Regulating the electronic structure of the Co–O framework is therefore an effective route for improving high-voltage stability (Kong et al., 2021). Recent studies have shown that LiCoO_2_ charged beyond 4.6 V undergoes complicated structural evolution, indicating that high-voltage performance should be evaluated not only by initial discharge capacity but also by the reversibility of oxygen and transition-metal frameworks (Wu et al., 2023). In this sense, the future of LiCoO_2_ lies in controlled lattice activation rather than simple voltage increase.

Surface chemistry is another key factor. In sulfide-based solid-state systems, for example, the contact between LiCoO_2_ and sulfide electrolytes can trigger interfacial reactions, which makes surface-base regulation and protective layers necessary (Yang K. et al., 2023). This indicates that cathode modification should be designed according to the electrolyte environment. A coating that works in a carbonate liquid electrolyte may not automatically be effective in a sulfide or oxide solid-state battery. Thus, interfacial compatibility should become a central criterion when designing high-voltage LiCoO_2_.

Elemental doping has been widely used to improve lattice stability. Trace Er and Mg co-doping has been reported to stabilize LiCoO_2_ at 4.6 V by regulating different lattice sites, showing that site-specific doping is more meaningful than random element addition (Xia et al., 2024). Mg-pillared LiCoO_2_ further illustrates that suitable dopants can suppress unfavorable phase transitions and improve high-voltage cycling (Huang et al., 2021). These results suggest that dopants should be selected according to their role in oxygen stabilization, lithium diffusion, and stress relaxation.

Surface coating provides another level of protection. Nanoscale solid-electrolyte coatings can improve interfacial stability while maintaining lithium-ion transport (Li et al., 2020). Polyanionic species anchoring has also been shown to enhance reaction kinetics and structural stability by modifying surface oxygen activity and lithium diffusion behavior (Zheng et al., 2024). More recently, near-surface layer regulation has been used to suppress intergranular cracking and improve electrochemical–thermal stability (Song et al., 2025). These strategies show that the most promising LiCoO_2_ design is not a single coating or single dopant, but a coordinated surface–subsurface architecture that stabilizes oxygen, protects the electrolyte interface, and preserves particle integrity.

## 3 Spinel LiMn_2_O_4_: Suppressing Mn dissolution and Jahn–Teller-driven instability

Spinel LiMn_2_O_4_ is attractive because manganese is abundant, low-cost, and less toxic than cobalt. Its three-dimensional lithium-ion diffusion network offers good rate capability, making it suitable for power-oriented applications. The spinel framework also has historical importance in lithium-ion battery development (Thackeray, 2021). However, LiMn_2_O_4_ has long been limited by capacity fading, especially under elevated temperature, high rate, or extended cycling. The main degradation pathways include Mn dissolution, Jahn-Teller distortion associated with Mn^3+^, surface reconstruction, and electrolyte-induced side reactions (Hou et al., 2023).

Unlike LiCoO_2_, where high-voltage oxygen instability is often the central issue, LiMn_2_O_4_ suffers from a more pronounced coupling between transition-metal chemistry and electrolyte attack. Mn^3+^ disproportionation can generate soluble Mn^2+^, which migrates into the electrolyte and may deposit on the anode. This process not only consumes active manganese but also disrupts the spinel framework. Therefore, stabilizing Mn valence and reducing direct electrolyte contact are two key strategies for improving LiMn_2_O_4_ durability.

One emerging direction is to engineer defects or grain boundaries intentionally rather than simply eliminating them. Ultrafast nonequilibrium processing has been used to introduce twin boundaries into spinel lithium manganate, improving structural stability and electrochemical behavior (Guo et al., 2024). This suggests that controlled nonequilibrium structures may provide fast transport pathways or stress-buffering regions. Instead of viewing defects only as harmful, future LiMn_2_O_4_ design may use selected defects as functional structural units.

Cation substitution is another important approach. First-principles studies of Ni-doped spinel Mn-based cathodes indicate that Ni substitution can modify lithium diffusion behavior, reduce Jahn–Teller distortion, and improve discharge characteristics (Li et al., 2023). This is especially important because LiMn_2_O_4_ cannot achieve long-term stability only by surface protection; the internal Mn–O framework must also be strengthened. In this respect, doping should be understood as a framework-stabilization strategy.

Surface engineering also plays a decisive role. *In situ* LaBO_3_ coating has been used to form a protective surface and regulate particle morphology, suppressing Mn dissolution and improving cycling stability (Chen et al., 2024). Surface Mg substitution at the 8a tetrahedral sites has also been reported to suppress manganese dissolution and enhance cycle stability without heavily sacrificing capacity (Yang S. et al., 2023). These examples show that LiMn_2_O_4_ improvement requires both internal and external control: internal substitution reduces structural distortion, while surface engineering limits electrolyte attack and transition-metal dissolution.

From a practical viewpoint, LiMn_2_O_4_ is unlikely to compete with Ni-rich layered oxides in maximum energy density. Its opportunity lies instead in cost-sensitive, safety-sensitive, and high-power applications. Therefore, future research should not only report half-cell capacity retention, but also examine full-cell Mn crossover, thermal stability, electrolyte compatibility, and calendar aging. A failure-mode-oriented approach may help LiMn_2_O_4_ regain importance in applications where low cost, high power, and moderate energy density are more valuable than maximum capacity.

## 4 LiFePO_4_: From nanoscale kinetics to thick-electrode utilization

LiFePO_4_ is one of the most successful cathode materials for safe and long-life lithium-ion batteries. Its olivine framework provides excellent thermal stability and strong P–O bonding, which makes it safer than many layered oxide cathodes. However, LiFePO_4_ also has intrinsic limitations, including low electronic conductivity and one-dimensional lithium-ion transport. Carbon coating, particle size reduction, and morphology control have therefore been central themes in LiFePO_4_ research (Chen et al., 2022; Eftekhari, 2017).

### 4.1 Particle-level kinetics and surface conductive modification

At the particle level, nanosizing, carbon coating, morphology regulation, and elemental doping have been widely adopted to improve LiFePO_4_ kinetics. In early LiFePO_4_ studies, nanosizing was considered an effective strategy because smaller particles shorten lithium-ion diffusion length and increase the electrochemically active surface area. However, this approach also has trade-offs. Extremely small particles can increase the electrode–electrolyte contact area, accelerate parasitic reactions, increase carbon and binder demand, and reduce tap density. Therefore, particle-size optimization should not be understood as simply making LiFePO_4_ particles as small as possible, but as balancing ion transport, electronic conduction, surface stability, and electrode packing density.

Surface conductive modification remains a central strategy for LiFePO_4_. *In situ* carbon coating using polymer-derived carbon sources can form a relatively uniform electronic-conductive layer and improve cycling performance (Shi et al., 2021). Hybrid coatings that combine carbon with ion-conductive components, such as Li_1.3_Al_0.3_Ti_1.7_(PO_4_)_3_, further indicate that future LiFePO_4_ modification may require both electronic and ionic transport functions rather than simple electronic conductivity alone (Nguyen et al., 2023). In addition, La and Ce co-doping has been reported to improve the rate performance of LiFePO_4_ by modifying lithium transport and structural behavior (Syed et al., 2024). These studies show that particle-level modification is still important, but its practical value depends on whether improved local kinetics can be retained in high-loading electrodes.

### 4.2 Electrode-level LiFePO4 design: Thick electrodes and dry processing

Beyond particle-level kinetics, LiFePO4 increasingly highlights the importance of electrode-level engineering. For practical batteries, the limiting factor is not only the intrinsic conductivity of individual particles, but also the continuity of the conductive network, lithium-ion transport tortuosity, binder distribution, electrode porosity, interparticle contact resistance, and compaction density. These electrode-level factors become more critical when the areal loading and electrode thickness increase.

Recent work on dry-processed thick LiFePO_4_ electrodes clearly reflects this transition from material-level optimization to electrode-level design. Low-resistance thick-film electrodes prepared by dry electrode technology demonstrate that LiFePO_4_ performance can be improved by constructing efficient conductive networks, reducing inactive components, and maintaining mechanical integrity under high loading (Kwon et al., 2024). Similarly, large-particle LiFePO_4_ has been investigated for high energy density, suggesting that larger particles are not necessarily disadvantageous if their morphology, packing behavior, and conductive environment are properly controlled (Syed et al., 2024). More broadly, recent dry-processed thick-electrode studies show that high areal capacity can be achieved by optimizing conductive-agent morphology, electrode density, and transport pathways, confirming that electrode architecture is becoming a decisive factor for practical high-energy-density lithium-ion batteries (Oh et al., 2025).

Therefore, the key innovation direction for LiFePO_4_ is shifting from “faster particles” toward “better electrodes.” A practical LiFePO_4_ cathode should combine stable particles, continuous electronic pathways, efficient lithium-ion transport channels, low binder resistance, appropriate porosity, high compaction density, and scalable manufacturing compatibility. From a failure-mode-oriented perspective, LiFePO_4_ is a representative example showing that mature cathode materials can still be improved through electrode architecture optimization rather than only through nanosizing, doping, or surface coating.

## 5 Ni-rich layered oxides: controlling oxygen activity, cation disorder, and particle cracking

Ni-rich layered oxides such as NCM811 and NCA are among the most promising high-energy cathodes because increasing Ni content raises reversible capacity and reduces cobalt dependence. However, higher Ni content also introduces severe instability. Ni-rich cathodes are sensitive to cation mixing, residual lithium compounds, surface reconstruction, oxygen release, electrolyte oxidation, and intergranular cracking (Jiang et al., 2021; Julien and Mauger, 2020). These issues are not isolated; they reinforce one another during cycling.

Recent advances in Ni-rich cathode materials have highlighted that achieving stable high-energy performance requires simultaneous optimization of composition, microstructure, and interfacial chemistry. Although increasing Ni content improves capacity, it also intensifies oxygen instability, cation disorder, surface reconstruction, and mechanical degradation. Therefore, the design of Ni-rich cathodes should focus on balancing energy density and structural durability rather than simply maximizing Ni concentration (Fan et al., 2023; Wu et al., 2024).

Oxygen redox and transition-metal dissolution are central degradation pathways in Ni-rich cathodes. When highly delithiated, the lattice becomes more oxidizing, and oxygen instability can promote surface phase transformation and metal dissolution (Cai et al., 2023). Operando studies of LiNi_0.8_Co_0.1_Mn_0.1_O_2_ have further revealed that chemo-mechanical evolution is a critical factor in cycling degradation (Zhang et al., 2024). This means that Ni-rich cathodes must be designed not only for high initial capacity but also for mechanical reversibility during repeated lattice breathing.

Super Ni-rich and Co-poor cathodes are especially attractive for cost and energy-density reasons, but their development requires more precise control of composition, microstructure, and interface chemistry (Mubarac et al., 2024). Ni-rich/Co-poor materials offer high promise for automotive applications, but they also face challenges in safety, air stability, moisture sensitivity, and long-term durability (Wang et al., 2020). Therefore, the most realistic future path may not be simply pushing Ni content as high as possible, but identifying a balance between capacity, processability, and structural robustness.

Surface coatings can reduce direct contact between cathode particles and electrolyte. For example, Li_1.3_Al_0.3_Ti_1.7_(PO_4_)_3_ fast-ion-conductor modification of NCA improves electrochemical performance by providing a protective and ionically conductive surface (Nie et al., 2020). Gradient coatings and concentration-gradient structures are also useful because they maintain a high-Ni core for capacity while using a more stable surface composition to resist electrolyte attack (Song et al., 2024). This core–shell logic is particularly suitable for Ni-rich cathodes because the internal and external parts of the particle face different requirements (Wang and Meng, 2023).

In addition to single surface modification, recent studies have demonstrated that the combination of bulk doping and surface coating can provide synergistic stabilization effects for Ni-rich cathodes. For example, the coupling of F^−^ doping and fluorocarbon coating in NCM811 can simultaneously strengthen the bulk structure and regulate the cathode–electrolyte interface, thereby improving high-voltage cycling stability. Such combined strategies highlight the importance of integrating lattice stabilization with interfacial protection (Sun et al., 2025).

Recent 2025 studies further indicate that Ni-rich layered oxides are moving toward single-crystal, entropy-stabilized, and surface-reconstructed designs. Compared with conventional polycrystalline secondary particles, single-crystal Ni-rich cathodes can reduce intergranular crack formation and limit electrolyte penetration along grain boundaries, which is particularly important under high-voltage or fast-charge conditions. Meanwhile, high-entropy doping introduces multiple cations into the transition-metal framework or near-surface region, aiming to reduce irreversible phase transition, stabilize metal–oxygen coordination, and suppress crack propagation. Such strategies shift Ni-rich cathode design from simple composition optimization toward coupled lattice, surface, and mechanical stabilization. However, their practical value should be evaluated not only in coin-type half cells but also in high-loading full cells, where electrolyte oxidation, transition-metal crossover, gas evolution, and impedance accumulation become more visible (Pandey et al., 2025; Zhou et al., 2025).

Doping is equally important. Doping strategies for NCM cathodes aim to suppress cation disorder, strengthen transition-metal–oxygen bonds, improve lithium diffusion, and reduce structural collapse (Ko et al., 2023). Ti incorporation into Ni-rich layered cathodes is a representative example, because strong Ti–O bonding can improve particle integrity and reduce crack formation during cycling (Kim et al., 2020). In future studies, dopants should be selected according to their role in lattice stabilization, oxygen control, and mechanical reinforcement, rather than only according to capacity retention in short-term cycling.

## 6 Toward integrated cathode design: coupling structure, interface, and mechanics

To further connect cathode degradation pathways with practical design strategies, Table 1 summarizes the main failure modes and targeted modification routes for representative lithium-ion battery cathodes.

**TABLE 1:** Failure-mode-oriented design strategies for representative lithium-ion battery cathodes.

Cathode system	Main failure mode/limitation	Targeted design strategy	Practical goal
High-voltage LiCoO_2_	Oxygen loss, surface reconstruction, microcracking	Trace doping, polyanion anchoring, protective coating	Stable high-voltage cycling
Spinel LiMn_2_O_4_	Mn dissolution, jahn–Teller distortion, capacity fading	Cation substitution, defect regulation, surface coating	Durable low-cost power cathode
Olivine LiFePO_4_	Low conductivity and limited Li^+^ diffusion	Carbon coating, particle engineering, hybrid conductive coating	Safe long-life cathode with improved rate capability
Thick-electrode LiFePO_4_	High-loading transport resistance and polarization	Dry electrode processing and conductive-network optimization	Practical high-energy-density electrodes
Ni-rich layered oxides	Cation mixing, oxygen instability, particle cracking	Bulk doping, gradient coating, mechanical stabilization	High energy density with controlled degradation

### 6.1 Electrolyte-dependent interface chemistry and full-cell crosstalk

Electrolyte-dependent interface chemistry is a critical bridge between cathode surface degradation and practical cell failure. For high-voltage LiCoO_2_ and Ni-rich layered oxides, carbonate electrolyte oxidation can produce unstable CEI components, gaseous products, and acidic species derived from LiPF_6_ decomposition. These products further promote surface reconstruction, oxygen release, and transition-metal dissolution. Therefore, the cathode surface should not be evaluated as an isolated solid surface, but as a dynamic reaction interface determined by the cathode potential, electrolyte solvation structure, salt chemistry, additive decomposition pathway, and operating temperature (Jin et al., 2025).

Fluorinated solvents, film-forming additives, localized high-concentration electrolytes, and nitrile- or phosphate-containing additives have been explored to construct more stable CEI layers. A desirable CEI should be electronically insulating, ionically conductive, chemically resistant to high-voltage oxidation, and mechanically robust during lattice breathing. Inorganic-rich or F-rich interphases can suppress continuous electrolyte decomposition and reduce transition-metal dissolution, but excessive or nonuniform CEI growth may increase impedance and aggravate local polarization. Therefore, electrolyte design should be coordinated with cathode coatings and surface reconstruction rather than treated as a separate cell component.

Full-cell validation is also essential. Li-metal half cells are useful for screening cathode activity, but excess lithium can compensate for lithium-inventory loss and obscure anode-side degradation. In practical graphite- or thin-Li-based full cells, dissolved transition-metal ions can migrate to the anode, catalyze electrolyte decomposition, thicken the SEI, increase polarization, and induce anode slippage. These crosstalk effects are especially relevant for Ni-rich cathodes operated at high voltage. Therefore, future studies should report not only half-cell capacity retention, but also full-cell cycling under practical areal loading, limited electrolyte, controlled N/P ratio, *postmortem* transition-metal deposition analysis, gas evolution, and impedance evolution (Yang et al., 2025).

### 6.2 Multi-level integrated cathode design: coupling lattice, interface, mechanics, and electrode architecture

The four cathode families discussed above show different advantages, but their degradation mechanisms share several common features. High-voltage LiCoO_2_ suffers from oxygen instability and interfacial side reactions; LiMn_2_O_4_ is limited by Mn dissolution and Jahn–Teller distortion; LiFePO_4_ requires improved ion/electron transport in practical thick electrodes; Ni-rich oxides face cation disorder, oxygen release, and particle cracking. These differences suggest that no universal modification strategy exists. However, a universal design philosophy can still be proposed: cathode materials should be engineered according to their dominant failure modes.

First, surface modification should be designed as a functional interphase, not merely as a passive coating. A useful cathode surface layer should block parasitic reactions, allow lithium-ion transport, remain chemically compatible with the electrolyte, and tolerate volume change. This is why solid-electrolyte-like coatings, polyanionic anchoring, and ion-conductive surface layers are becoming increasingly important.

Second, bulk doping should be connected with clear structural functions. In LiCoO_2_, dopants can stabilize lattice oxygen and suppress high-voltage phase transition. In LiMn_2_O_4_, substitution can reduce Jahn–Teller distortion and Mn dissolution. In Ni-rich cathodes, dopants can reduce cation mixing, strengthen metal–oxygen frameworks, and limit crack formation. This means that future papers should provide stronger mechanistic evidence for why a dopant works, rather than simply reporting improved cycling data.

Third, mechanical stability should be treated as a core electrochemical parameter. Cathode particles expand, contract, crack, and reconstruct during cycling. Once cracks form, fresh surfaces are exposed to the electrolyte, which accelerates impedance growth and interfacial degradation. This problem is particularly serious in high-voltage LiCoO_2_ and Ni-rich layered oxides. Therefore, morphology regulation, grain-boundary engineering, concentration gradients, and surface–subsurface reconstruction should be considered as mechanical design tools.

Fourth, practical electrode conditions should be emphasized. Many cathode materials show good performance in low-loading half cells, but practical cells require high areal loading, thick electrodes, low inactive content, high compaction density, and stable full-cell operation. LiFePO4 studies using thick dry electrodes and large particles show that electrode architecture may be as important as material chemistry. Similar logic should be extended to layered oxides and spinel cathodes.

Recent studies further demonstrate that cathode durability should be evaluated beyond conventional half-cell testing. Under practical conditions, increased electrode thickness and active material loading can amplify ionic transport limitations, local polarization, and mechanical stress accumulation. Therefore, the optimization of cathode materials should be integrated with electrode architecture design to achieve balanced energy density and long-term stability.

## 7 Conclusions and outlook

Cathode materials for lithium-ion batteries have entered a stage where simple capacity improvement is no longer sufficient. The next-generation of cathode research should focus on failure-mode control under realistic cell conditions. For LiCoO_2_, the central issue is how to extract more lithium at high voltage while stabilizing oxygen, surface chemistry, and particle integrity. For LiMn_2_O_4_, the key task is to suppress Mn dissolution and Jahn–Teller distortion while preserving its low-cost and high-power advantages. For LiFePO_4_, the main challenge is not only intrinsic conductivity but also high-loading electrode architecture and scalable processing. For Ni-rich layered oxides, the critical goal is to balance high capacity with oxygen stability, reduced cation disorder, and resistance to microcracking.

Future cathode development should therefore integrate four levels of design. At the atomic level, dopants should be chosen to regulate oxygen activity, metal–oxygen bonding, and lithium diffusion barriers. At the surface level, coatings should be chemically stable, ionically conductive, and mechanically compatible. At the particle level, morphology, grain boundary, and concentration gradients should be optimized to reduce stress concentration and crack propagation. At the electrode level, conductive networks, binder systems, porosity, thickness, and compaction density should be designed for practical energy density.

A failure-mode-oriented framework can help cathode research move beyond empirical modification. Instead of asking whether coating, doping, or nanosizing improves a material, researchers should ask which degradation pathway is being suppressed, whether the improvement remains effective in full cells, and whether the design is compatible with scalable manufacturing. Such a shift will be essential for developing cathode materials that are not only high-performing in laboratory tests, but also durable, safe, and economically viable in practical lithium-ion batteries.
